# Preferential Invasion by *Plasmodium* Merozoites and the Self-Regulation of Parasite Burden

**DOI:** 10.1371/journal.pone.0057434

**Published:** 2013-02-27

**Authors:** Douglas H. Kerlin, Michelle L. Gatton

**Affiliations:** 1 Malaria Drug Resistance and Chemotherapy Laboratory, Queensland Institute of Medical Research, Brisbane, Queensland, Australia; Kenya Medical Research Institute - Wellcome Trust Research Programme, Kenya

## Abstract

The preferential invasion of particular red blood cell (RBC) age classes may offer a mechanism by which certain species of *Plasmodia* regulate their population growth. Asexual reproduction of the parasite within RBCs exponentially increases the number of circulating parasites; limiting this explosion in parasite density may be key to providing sufficient time for the parasite to reproduce, and for the host to develop a specific immune response. It is critical that the role of preferential invasion in infection is properly understood to model the within-host dynamics of different *Plasmodia* species. We develop a simulation model to show that limiting the range of RBC age classes available for invasion is a credible mechanism for restricting parasite density, one which is equally as important as the maximum parasite replication rate and the duration of the erythrocytic cycle. Different species of *Plasmodia* that regularly infect humans exhibit different preferences for RBC invasion, with all species except *P. falciparum* appearing to exhibit a combination of characteristics which are able to self-regulate parasite density.

## Introduction

An ideal parasite must derive benefit without killing its host, at least until the parasite has an opportunity to reproduce. Parasites causing malaria invade host red blood cells (RBCs), mature and replicate within the RBC and subsequently kill these host cells as schizont stage parasites rupture, causing the lysis of the RBC and releasing merozoite stage parasites into the blood stream to start the process over. Asexual reproduction during the erythrocytic phase of the *Plasmodium* life cycle can thus result in an exponential increase in the number of parasites, which has the capacity to induce significant anaemia in their host. In the case of malaria in a naïve host, anaemia is a major cause of morbidity, and, potentially mortality, particularly if the host is malnourished, has pre-existing anaemia or co-infections that increase the immunological burden on the host [1]. The parasite therefore must balance achieving a suitably high reproduction rate while maximising the probability the host will survive until the parasite can achieve sexual stage transmission. From the host perspective, slowing the growth rate or limiting the total number of parasites in the early stages of infection may be key to providing sufficient time to develop a specific immune response [2].

There are at least four possible strategies that parasites can adopt to regulate their reproduction, reduce the burden of infection, and ensure survival without killing the host:

1. Limit the range of RBC age classes that are able to be invaded (preferential invasion of certain RBCs).
2. Reduce the maximum parasite replication rate (i.e. produce fewer merozoites per schizont).
3. Increase the time required to complete each erythrocytic cycle.
4. Rely on the host immune response to control the parasite burden.

The preferential invasion of particular RBC age classes is characteristic of some species of human malaria parasites. *Plasmodium falciparum* is capable of invading all RBC age classes, while *P. vivax* and *P. ovale* demonstrate a strong preference for the youngest RBCs (reticulocytes) and *P. malariae* the mature RBCs [1], [3]. The RBC invasion preferences (if any) for *P. knowlesi* are still to be identified. It is generally accepted that *P. falciparum* is predominantly responsible for cases of severe disease and malaria-related mortalities, while the other human *Plasmodium* species, which are more discerning in the RBCs they invade, are relatively more benign disease agents [4]. While the severe complications associated with *P. falciparum* are not generally directly related to anaemia, the ability to achieve high parasite densities facilitates the development of conditions such as cerebral malaria [1], [4]. However, there are currently limited data on parasite dynamics and the loss of RBCs following infection in humans [5].

It has previously been posited that there is a relationship between disease severity and the age classes of erythrocytes infected, such that parasites which only target particular age classes are less likely to be associated with severe disease [6]. A preference for invasion of a limited selection of RBCs by merozoites (either through specific targeting of particular age classes or because other potential host cells are more difficult to invade [2]) may increase competition between merozoites for suitable host cells and will ultimately limit parasite numbers, in a manner not dissimilar to logistic population growth. In natural populations, the logistic growth model posits that competition for limited resources places an artificial cap on abundance [7]. Similarly, a strong restriction to RBCs of a particular age class may diminish the pool of susceptible host RBCs, reducing the number of merozoites able to successfully find a new host cell, an idea previously proposed by a number of authors [5], [8], [9]. As a proportion of parasites are killed, unable to find a suitable RBC to invade, the virulence of an infection is diminished, allowing time for an appropriate specific immune response to be established [2].

Conversely, modelling work has suggested that depletion of the reticulocyte population by the unchecked growth of a *P. vivax* infection can be detrimental to the host and result in severe disease outcomes [10]. In the absence of a host immune response, the constant removal of reticulocytes shortly after their introduction into the system can be catastrophic; new RBCs are continually destroyed while the older RBCs they are intended to replace naturally senesce, leading to a reduction in RBC abundance in all age classes. This contrasts with a *P. falciparum* infection, where losses are spread across a wider range of RBC age classes, so no particular class is subjected to heavy depletion, and some new cells are able to survive and replace older RBCs naturally leaving the system.

Another consequence of preferential invasion that suppresses parasite abundance is that gametocyte abundance is also suppressed, reducing the probability of parasite transmission to a mosquito. However the relationship between preferential RBC invasion and reduced transmission is currently unknown and requires further investigation.

For a species such as *P. vivax*, which is increasingly recognised as a major contributor to the global burden of malaria [11], [12], understanding the implications of preferential reticulocyte invasion, mediated by highly specific interactions between reticulocytes and *P. vivax* merozoite surface proteins [13], [14], is critical to attempts to model the dynamics of infection, and to developing a better understanding of the disease. Indeed, current efforts to create a reliable system of culture are focussing on providing an intensive supply of reticulocytes to promote successful reinvasion [15]. It has also been shown that preferential invasion of reticulocytes can lead to a lower reticulocyte count [16], and that an increased reticulocyte population in pregnancy facilitates higher parasite burdens and leads to more clinical presentations [17].

In this study we use a mathematical model to investigate the implications of preferential RBC invasion for the development of a malaria infection. The key question is whether preferential invasion, in the absence of a specific host immune response, can regulate an infection. We examine whether RBC invasion preferences in some *Plasmodium* species could self-limit the parasite burden, making the parasite less reliant on the host immune response to maintain their infection and transmission potential. We also examine the role of the parasite replication rate, both with respect to the number of new merozoites produced during each erythrocytic cycle and the speed with which the erythrocytic cycle is completed, in limiting the growth of the parasite population during an infection.

## Methods

**Figure 1:**
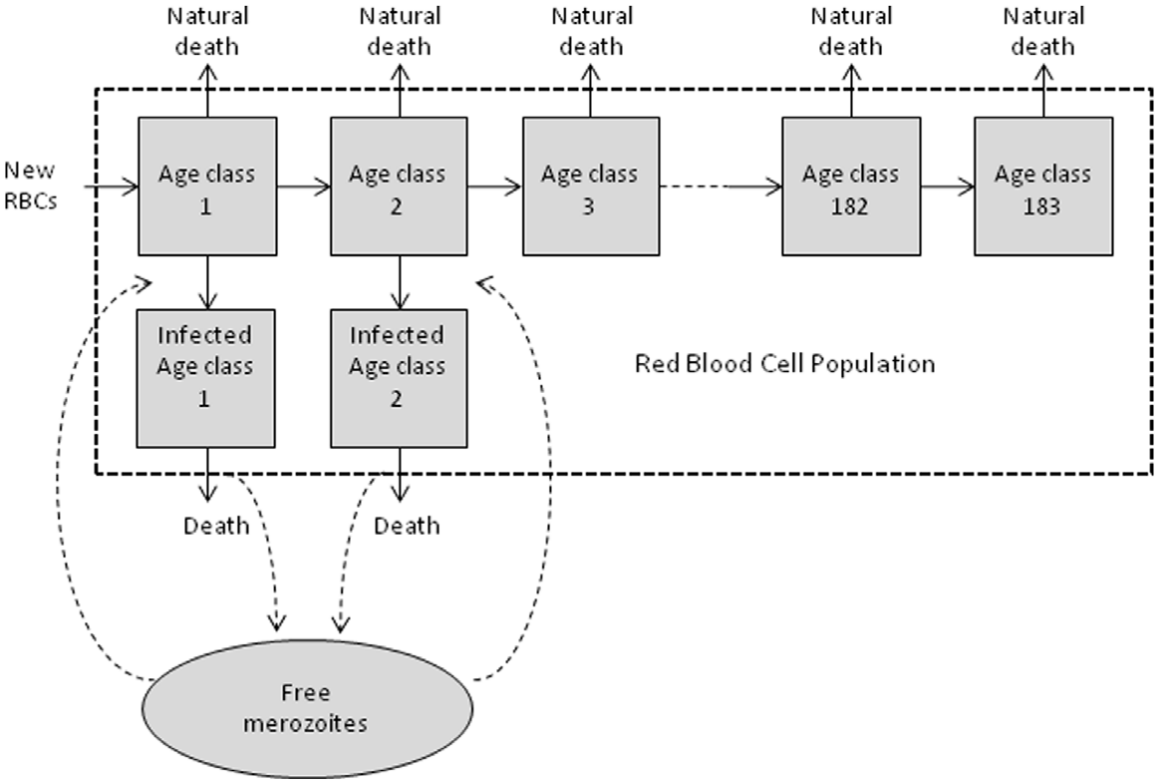
Conceptual model demonstrating the age structured model of host RBCs, with preferential invasion of age classes 1 and 2.

**Figure 2:**
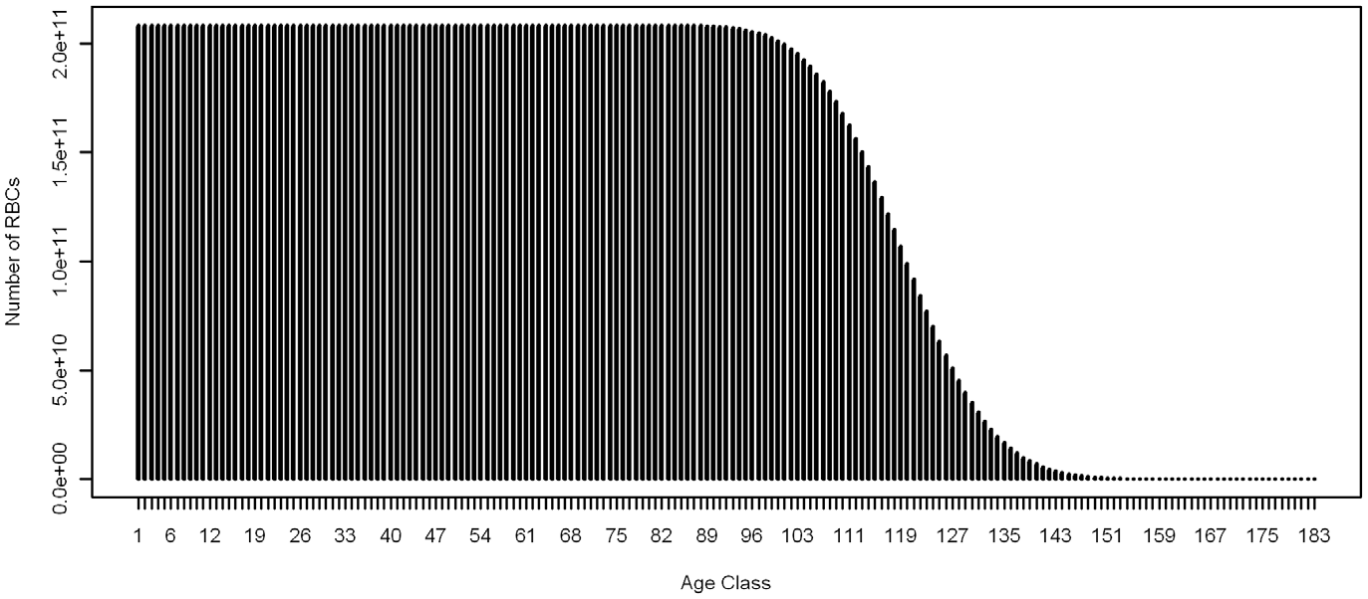
Age class structure of host RBC population prior to infection.

We developed a discrete-time simulation model of RBC dynamics, and introduce a modelled parasitic infection (Figure 1). As our starting point, we assumed the total starting RBC abundance is 2.5 × 10^13^, based on an average volume of 5 L of blood and 5 × 10^9^ RBCs/µL of blood [18]. RBC longevity is assumed to be Poisson distributed, with a mean (λ_d_) of 120 days [18], [19]. A Poisson distribution is used as it provides non-negative, approximately normally distributed (where *λ* is greater than 10) discrete values, and is preferred over other possible distributions due to the requirement for only one parameter which is reported in the literature. This provides a RBC age distribution with age classes from 1 to 183 days (Figure 2). The maximum age class was 183 days as the cumulative Poisson distribution function reaches unity beyond this age.

The model has a one day time-step with RBCs in age class *j* progressing to age class *j*+1. On each day we assume that the number of new reticulocytes (age class 1) is drawn from a Poisson distribution with mean λ_b_ = 2.5 × 10^13^/120 = 2.08 × 10^11^, sufficient to replace the older RBCs being removed through natural senescence each day. In this way the RBC abundance is at equilibrium in the disease-free state. Older RBCs are removed at each time step at a rate appropriate to maintain the Poisson structure of the population (i.e. for each age class *j*, the number of RBCs removed is determined by the proportional differences in the cumulative Poisson distribution for *j* and *j*-1).

We do not incorporate any defined specific host immune response to the parasite, nor do we initially incorporate any erythropoietic increase in RBC production in response to infection; instead, we are interested in the potential for intra-parasite competition for susceptible RBCs to limit parasite density. Similarly, we only remove RBCs after naturally occurring senescence and after destruction caused by the release of the schizont stage parasite (Figure 1).

An infection is introduced into this system by assuming that 10,000 merozoites are released from a single hepatic schizont. This value encompasses the documented numbers of released merozoites which range from 30,000 in *P. falciparum* to 2,000 in *P. malariae* [1]. Merozoites are released into the blood stream, and in reality have a limited amount of time to find a susceptible cell before they are removed from circulation [20], [21]. To mimic this we assume that the probability that a merozoite successfully invades a suitable RBC is initially dependent on the probability that RBCs are encountered, and having encountered a RBC, the probability that the encountered cell belongs to a susceptible age class. Consequently, we assume that the probability of invasion falls quadratically, rather than linearly, as the total RBC abundance, and the number of susceptible RBCs declines. We also incorporate a simplified, time-dependent reduction in merozoite survival to mimic basic clonal immunity. Probability of invasion success is thus assumed to be a function of:

1. Relative RBC density, defined as the total RBC population at time *i* 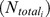 as a proportion of the total RBC population prior to infection 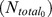, 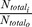,
2. The proportion of susceptible RBCs (the number of RBCs in susceptible age classes) available at time *i* 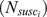, as a proportion of susceptible RBCs prior to infection 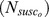,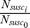,
3. Time-dependent reduction in merozoite survival (*c_i_*) which occurs as generic clonal immunity to the parasite develops during prolonged infections in the host: 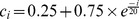. Parameters of this component of the model were arbitrarily selected to reduce the proportion of surviving merozoites from 1 to 0.25 over time.

Combining these components, the number of merozoites which successfully invade RBCs is determined by drawing from the Binomial distribution 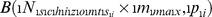, where 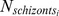 is the number of schizont stage parasites rupturing on day *i* and releasing merozoites, *m_max_* is the maximum potential number of merozoites released per schizont, and *p_i_* is the probability of successful invasion on day *i*:
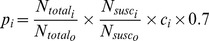



The scaling factor of 0.7 provides an adjustment from the biological maximum number of merozoites released (*m_max_*), to the actual mean number of merozoites which usually develop and invade a new RBC in naïve hosts.

Any free merozoites that do not successfully invade a susceptible RBC are assumed to have died and are removed from further consideration. Critically, if the number of merozoites which successfully invade RBCs is greater than the number of available susceptible RBCs, it is assumed that the number of merozoites which develop to mature schizonts is equal to the available susceptible RBCs. While a consequence of competition for a limited supply of susceptible RBCs may be invasions of a single RBC by multiple merozoites, the viability of these parasites is unknown. Multiply infected RBCs are sometimes observed under *in vitro* culture conditions, however there is little evidence of such events regularly occurring in natural *in vivo* infections. Even if multiple infections of single RBCs do occur *in vivo*, it is unclear whether both parasites would successfully reproduce to their maximum potential under such conditions. We therefore assume that while multiple infection of a single RBC is an option, it will only result in the release of new free merozoites equivalent to the number released from a single parasite.

The model was used to estimate changes in relative host RBC density during simulated infections with hypothetical malaria parasites. Two sets of simulations were conducted; the first assumed a 48 hour parasite erythrocytic cycle, as observed in *P. falciparum*, *P. ovale*, and *P. vivax*, while the second assumed a 72 hour parasite erythrocytic cycle as observed in *P. malariae.* The effect of preferential invasion on RBC dynamics was assessed by limiting the range of RBC age classes able to be invaded. This was achieved using two different approaches:

1. Preferential invasion of young RBCs. The maximum age of susceptible RBCs which could be invaded was increased (in one day increments) from 1 day old RBCs (*j = *1) to 150 day old RBCs (*j* = {1, 2,…,150}).
2. Preferential invasion of older RBCs. The minimum age of invaded RBCs was decreased (in one day increments) from 150 days (*j* = {150, 151,… 183}), to allowing invasion of all RBCs (*j = *{1,…149, 150,…183}).

Simulations were conducted for all combinations of *m_max_* (from 1 to 32) and age class sets, with three simulations conducted per combination, and average output values recorded. Simulated infections were followed for 400 days. Preliminary simulations indicated that some combinations of factors resulted in cyclic dynamics, but that the majority of these simulations reached an equilibrium within 400 days (Figure S1; Figure S2), though analysis of infections followed for 100 and 200 days provide nearly identical trends. Primary models output was minimum RBC abundance, an indication of the peak severity of anaemia. Model output was also recorded for 1) maximum parasite burden, 2) time required to reach maximum parasite burden, and relative RBC density at 400 days, representative of RBC loss in the patient, and calculated as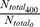.

The role of erythropoiesis in malaria infection is somewhat uncertain. While some have reported that erythropoiesis is up-regulated during a malaria infection to compensate for the loss of RBCs [22], [23], others have suggested that decreased erythropoiesis (low red blood cell production) is characteristic of severe malarial anaemia [24], [25], [26], and may be a host mechanism to protect against anaemia [8]. To this end we repeated the simulations, incorporating an erythropoietic response to test the model’s sensitivity to rates of RBC production. The number of additional reticulocytes recruited into age class 1 through up-regulated erythropoiesis (*N_add_*) was assumed to be a function of total RBC count, and that there was a lag of 6 days between stimulation and availability of these new reticulocytes [27]:
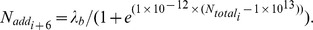



We did not consider the reverse situation where malaria infection may suppress erythropoiesis. This was due to uncertainty regarding the exact mechanism by which this may occur and the lack of published data suitable to derive model parameters.

The model was developed, and all analyses were conducted, using the R statistical computing software package [28]. Correlations between model outputs were examined using the Pearson product-moment correlation co-efficient.

## Results

Simulations were conducted across a range of scenarios (combinations of *m_max_* and susceptible age class sets) for both 48 and 72 hour erythrocytic cycles. For each simulation, a number of output variables were recorded: minimum RBC abundance, peak parasitemia, time to reach peak parasitemia, and relative RBC density at 400 days. There was a significant inverse correlation between minimum RBC abundance and peak parasitemia for results of all scenario models with an erythrocytic cycle duration of 48 hours examining preferential invasion of young RBCs (*r* = −0.629, *p*<0.001), and also those examining preferential invasion of older RBCs (*r* = −0.94, *p*<0.001). A similar relationship was apparent for scenarios assuming an erythrocytic cycle duration of 72 hours, (*r* = −0.944, *p*<0.001). Hence, the higher the burden of infection, the more severe the loss of red blood cells.

Further, there were significant inverse correlations between minimum RBC abundance and relative RBC density at 400 days for all three simulated data sets (48 hour erythrocytic cycle duration examining preferential invasion of young RBCs, 48 hour erythrocytic cycle duration examining preferential invasion of older RBCs and 72 hour erythrocytic cycle duration examining preferential invasion of older RBCs; r = −0.912, p<0.001; r = −0.944, p<0.001; r = −0.973, p<0.001 respectively), and between peak parasitemia and time to reach peak parasitemia (i.e. higher peak parasitemias were reached more quickly than lower peak parasitemias; r = −0.292, p<0.001; r = −0.139, p<0.001; r = −0.163, p<0.001 respectively). All of these comparisons were significant with an appropriate Bonferroni correction.

**Figure 3:**
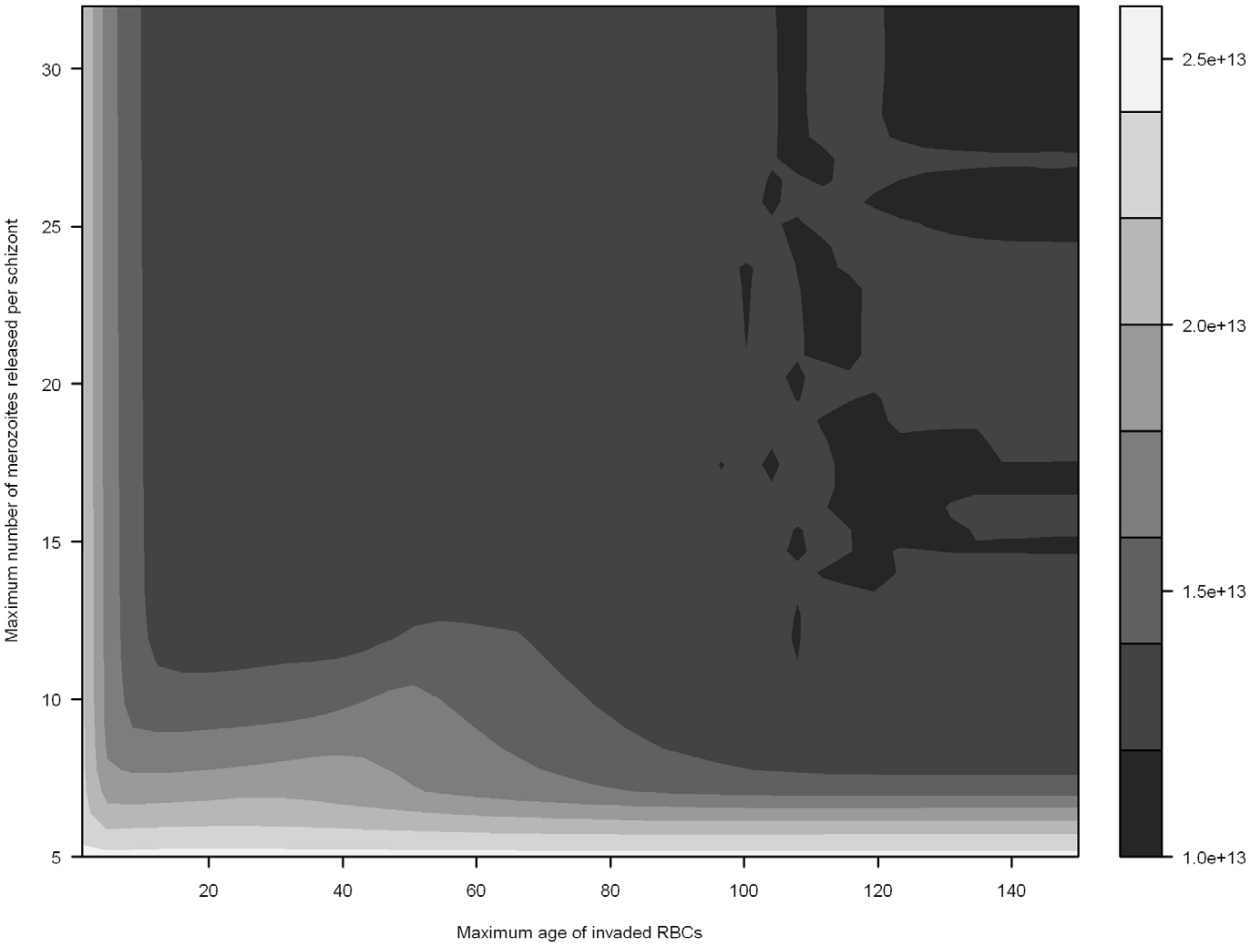
Preferential invasion of young RBCs and minimum RBC abundance in infected hosts. Minimum RBC abundance is mapped as a function of the maximum number of merozoites released per schizont, and the incrementally increasing maximum age of susceptible RBCs (simulated with an erythrocytic cycle duration of 48 hours). Darker areas indicate greater RBC loss.

**Figure 4:**
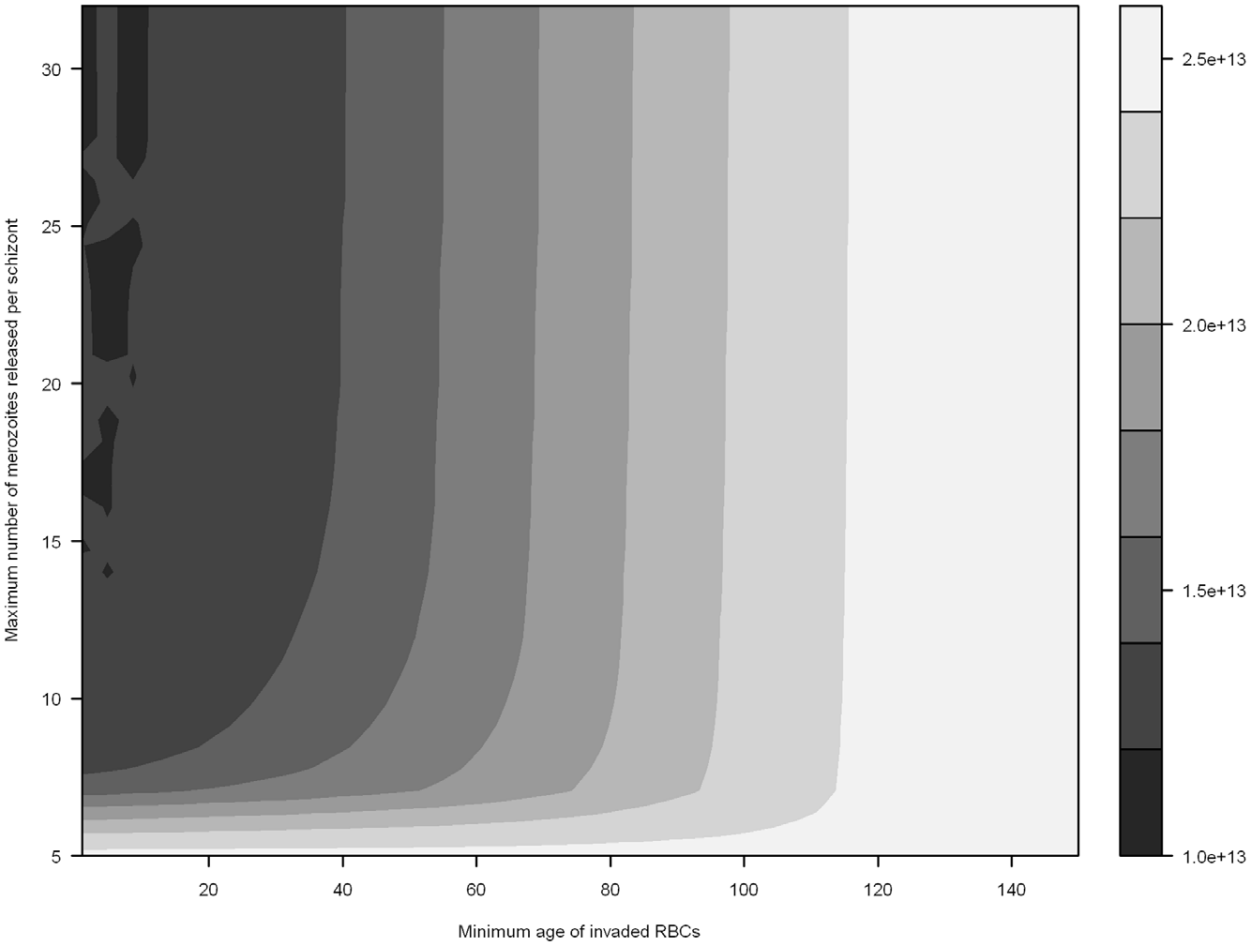
Preferential invasion of older RBCs and minimum RBC abundance in infected hosts. Minimum RBC abundance is mapped as a function of the maximum number of merozoites released per schizont, and the incrementally decreasing minimum age of susceptible RBCs (simulated with an erythrocytic cycle duration of 48 hours).

**Figure 5:**
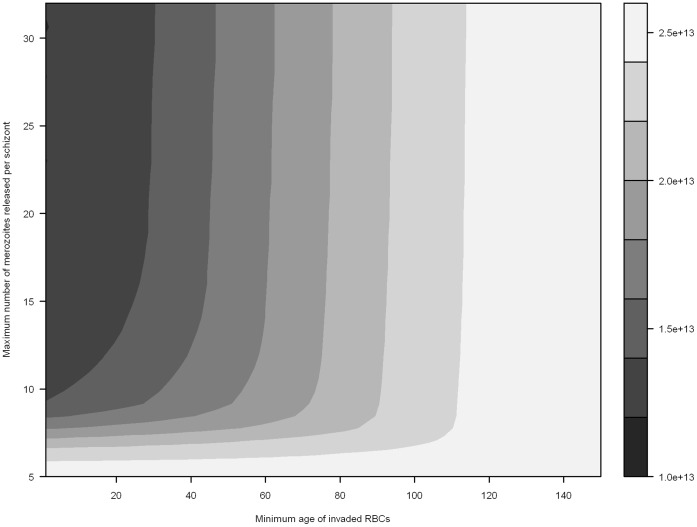
Preferential invasion of older RBCs and minimum RBC abundance under a 72 hour erythrocytic cycle duration. Minimum RBC abundance is mapped as a function of the maximum number of merozoites released per schizont, and the incrementally decreasing minimum age of susceptible RBCs.

Infections with a low multiplication rate (≤5) were typically not self-sustaining; if these infections were self-sustaining they had little impact on host RBC population (Figures 3, 4 and 5).

When *m_max_* exceeds 5 merozoites per schizont, our model results indicate that preferential invasion of young RBCs can have a profound impact on acute and long-term anaemic status; the larger the set of susceptible RBC age classes, the greater the associated RBC loss (Figure 3; Figure S3). Indeed, anaemia is generally more sensitive to the number of RBC age classes able to be invaded than the parasite replication rate. Where the maximum age of invaded RBCs is less than ∼10 days, a *m_max_* of 10 removes 33.6% of RBCs, while a *m_max_* of 30 only removes 37.3%; competition for a restricted number of susceptible cells reduces the advantage of producing large numbers of merozoites. Supplementary figures illustrate the impact of preferential invasion on other model outputs: peak parasitemia (Figure S4; the more age classes invaded, the greater the parasite burden) and the time required to reach maximum parasitemia (Figure S5; the more age classes invaded, the more time generally required to reach peak parasitemia).

Similar conclusions can be drawn from examining preferential invasion of older RBCs (Figure 4). Two consistent patterns emerge: 1) a low *m_max_* (<5) is associated with little anaemia, and 2) preferentially limiting invasion to a subset of older RBC age classes can temper anaemia associated with high values of *m_max_*. Further, it is generally apparent that invading older RBC age classes results lower RBC loss (in comparison in invading younger RBC age classes).

The importance of the duration of the erythrocytic cycle in defining the anaemic status was assessed using the model for preferential invasion of older RBCs. Comparing Figure 5 (72 hour erythrocytic cycle) with Figure 4 (48 hour erythrocytic cycle) shows that, on average, a 48 hour erythrocytic cycle is associated with an additional 7.789 × 10^11^ erythrocytes lost. A small proportion of simulations (0.01% of comparisons) had lower anaemia with a 48 hour cycle compared to a 72 hour cycle, but no consistent pattern is apparent; further simulations for each scenario would likely remove these peculiar results.

These results can provide insights into the human malaria parasite species since estimates for *m_max_* have been previously documented for the different human *Plasmodium* species in naïve hosts [1]. If we assume host death occurs when the RBC population is reduced by 50% (though recognising that a host would in reality likely die before this threshold were attained), applying these values to our results indicate that *P. falciparum* (*m_max_* = 32, 48 hour replication cycle and capable of invading all RBCs), in the absence of any additional immune response, will cause mass anaemia and kill the host within 20 days; the maximum parasite replication rate, and the unrestricted supply of RBCs provide ideal conditions for unconstrained growth of the parasite.

In comparison, *P. vivax* (*m_max_* = 18 and restricted to invading reticulocytes, RBC age class <2 days) infections do not kill the host within the 400 day timeframe, and only remove 15. 4% of total RBCs if there is no increased erythropoiesis. The results for *P. ovale* (*m_max_* = 14 and restricted to invading reticulocytes) are similar to *P. vivax*, with only 15.4% of RBCs removed after 400 days.

Examining where *P. malariae* fits in the spectrum of our results is difficult due to a paucity of specific data on the invasion preferences of the parasite; while a preference for older RBCs is well established in the literature [1], which age classes constitute ‘older RBCs’ has not previously been defined. It is clear from Figure 3, however, that given the low maximum parasite replication rate (*m_max_ = *10, 72 hour replication rate) that the host could survive for 400 days of infection, even if all non-reticulocyte RBC age classes are available for invasion. It is further apparent that invading a smaller subset of RBC age classes does result in a significant change in the anaemic status of the host. If we assume, for instance, that *P. malariae* only invades RBC age classes greater than 50 days, after 400 days we would only expect the removal of 22.3% of RBCs.

## Discussion

We have developed a simulation model to assess whether limiting the range of RBC age classes available for invasion is a credible mechanism for controlling the density of parasites, and thus anaemia. We were specifically interested in the ability of intra-parasite competition for susceptible RBCs to control parasite burden so elected not to include any specific host immune response in the simulation model. This includes the effects of fever or antibodies targeting specific parasite antigens. As all human malarias cause fever and other immune responses at sufficient parasite densities, the simple model does not reflect the full panel of factors influencing parasite density, instead presenting a worst case scenario where only parasite replication and invasion strategies impact the overall parasite burden. In adopting this approach we were able to determine whether the parasites were dependent on a host immune response for survival, or whether they were indeed self-limiting, creating the potential to exist for extended periods within the host. While the current study has focused on *Plasmodium* parasites, the general structure of the model is suitable for simulating any parasite which invades and destroys host RBCs.

From the simulation results it is clear that limiting the number of RBC age classes available for invasion, either by targeting younger or older RBCs, dramatically affects the progress of infection with regards to both the parasite density and host anaemic status. Limiting merozoite invasion to older RBCs as observed in *P. malariae* appears to be a more effective strategy than targeting reticulocytes (as observed in *P. vivax* and *P. ovale*), as the parasite is less likely to interfere with the recruitment of new RBCs; if a parasite targets reticulocytes, new RBC cohorts will suffer immediate losses, losses which accumulate as older, invasion resistant RBCs are replaced by new, susceptible, and thus numerically reduced cohorts [10]. Further, if erythropoiesis is indeed stimulated by a loss of blood cells, targeting only new RBCs may have perverse outcomes as the proportion of susceptible RBCs increases.

The model shows some interesting and somewhat unexpected results, namely that in situations where there is strong preferential invasion of young RBCs, the maximum parasite replication rate (*m_max_*) has little impact on acute and chronic anaemia. Although the available susceptible population is constantly replenished by new reticulocytes entering the system, the susceptible RBCs only represent a small proportion of the total RBCs and the parasites reach an equilibrium density quickly after infection due to the relatively constant, highly restricted number of susceptible RBCs. These external factors appear to have a greater impact on the overall system, compared to parasite replication rate. However, as the number of age classes suitable for invasion increase, parasite replication becomes an increasingly important factor.

Using a parameter set representative of *P. falciparum*, as the most lethal human malaria species, produces results which show this parasite to be consistently lethal in the absence of specific host immune response. That said, there is some recognition that *P. falciparum* may practise mild preferential invasion [29], [30]. It may be that *P. falciparum* does not explicitly require reticulocytes, but may still target relatively younger RBCs; certainly *P. falciparum* will invade mature RBCs, but it is unclear whether invasion is limited to a younger subset of mature RBCs. Studies of RBC selectivity suggest that *P. falciparum* selectivity is inversely proportional to disease severity [31], whereby high selectivity is observed at low parasitemia, but this declines at higher parasitemias; it has been speculated that *P. falciparum* may down-regulate its multiplication rate to avoid overwhelming its host [32], and changes to the parasites invasion preferences could provide a mechanism to allow this.

By comparison, *P. vivax* infection is self-limiting and although it does result in a loss of RBCs, this loss was not to the point where the host is threatened, at least after 400 days of infection. Limiting invasion to a small subset of available RBCs reduces the number of merozoites that successfully find and invade a new reticulocyte with the remaining free merozoites perishing before finding a suitable RBC. Given the reported 34–180 fold preference of *P. vivax* parasites for reticulocytes [2], and a development time for reticulocytes of 2 days, it is worth noting that *P. vivax* likely invades an even smaller subset of RBCs than generally acknowledged. Unless the parasite halts RBC development shortly after invasion, the parasite must target particularly young reticulocytes, such that by the conclusion of the erythrocytic cycle and subsequent lysis of the cell, the reticulocyte has not had sufficient time to develop into a mature RBC.

Preferential invasion by *P. ovale* and *P. malariae* parasites is far less well understood, due to the rare and benign nature of these infections. However, our results suggest that limiting invasion to reticulocytes improves the probability that a host survives *P. ovale* infection, as does the comparably low maximum parasite replication rate; fewer merozoites per schizont, and lower odds that these parasites successfully invade an appropriately aged RBC reduces the anaemic status induced in the host. Similarly, an infection with the *P. malariae* parasite, which adopts both a strategy of reducing the number of merozoites per schizont, and selective targeting of mature RBCs, can potentially last for up to 40 years without causing lethal anaemia [1]. A longer erythrocytic cycle improves the probability of parasite survival; with an erythrocytic cycle duration of 48 hours, the anaemic status of the host is compromised at a faster rate, generally irrespective of the maximum parasite replication rate, or preferential invasion, however these variables also play an important role. Parameters indicative of *P. falciparum* still result in the death of the host if we stipulate a longer erythrocytic cycle.

We have not considered *P. knowlesi*, a simian malaria parasite found in Southeast Asia that is capable of causing malaria in humans, due to the paucity of available information. However we can note that, given the 24 hour erythrocytic cycle reported for this species [1], the parasite might be expected to have the capacity to cause the rapid loss of RBCs and host death in the absence of an effective immune response. Infection with *P. knowlesi* is associated with high rates of severe disease, high case mortality and a variety of severe complications such as respiratory distress, renal failure and shock [33].

We have used anaemia, either acute (minimum RBC density) or chronic (relative RBC density after 400 days of infection) as the primary output of the model since it is easy to quantify, but recognise that in reality, very few patients actually die from acute anaemia from non-falciparum malaria infections, although severe malarial anaemia is the major cause of childhood deaths associated with *P. vivax* [12]. However anaemia resulting from haemolysis (including the haemolysis itself), and the subsequent fewer circulating RBCs can contribute to many of the complications associated with malaria such as hyperbilirubinemia and blackwater fever, and can exacerbate heart and lung complications. It is also worth noting that studies suggest that infection can result in a significant additional loss of uninfected RBCs as the immune system attempts to clear the infection [22]. Therefore we use the term anaemia as a surrogate for complications which are somehow related to the loss of RBCs.

The invasion preferences developed by human *Plasmodium* parasites have presumably evolved to create a beneficial environment for the parasite, one which provides ample opportunity for transmission. We hypothesise that the degree of evolution in self-limiting techniques such as preferential RBC invasion is related to the degree of concordant evolution of the parasite with its host, or rather, the time it has existed as a human pathogen. Phylogenetic studies suggest that *P. vivax* and *P. ovale* are closely related, with *P. malariae* placed in the same clade; in contrast *P. falciparum* is placed in a distant clade [34], [35]. Interestingly, *P. vivax* and *P. ovale* have almost identical host RBC invasion preferences, and the three species (*P. vivax*, *P. ovale* and *P. malariae*) have all developed some form of preferential invasion, whereas *P. falciparum* does not reliably exhibit a marked preference. It is not clear why *P. falciparum* has not developed such a feature; of the human malaria species, it certainly produces the largest number of merozoites, and thus would most benefit from self-regulatory actions to prevent host death prior to reproduction. Possible explanations may include highly stimulatory antigens in *P. falciparum* which elicit a strong host immune response that caps parasite density, or *P. falciparum* may be at a fitness disadvantage compared to *P. vivax* in competing for optimal RBCs in regions where mixed infections with these species are common, necessitating a more diverse invasion profile. While preferential RBC invasion appears to have benefits for an individual parasite, mixed infections where both species are competing for the same host cell would in fact be detrimental for both parasites. It is likely that some combination of these and/or other factors may play some role in defining the invasion preferences for a parasite, but there is insufficient data available to determine which are important.

The model we have developed does have a number of limitations that deserve mention. In determining to focus on the infection limiting role of preferential invasion, we have not incorporated any defined specific host immune response to the parasite, which may in fact play the predominant role in limiting parasite burden. Similarly, we only remove RBCs after naturally occurring senescence and after infection and subsequent rupturing, despite evidence that a significant number of uninfected erythrocytes are also destroyed during malaria infection [22], [36]. We have also assumed that multiple parasites invading a single RBC will not all produce more viable merozoites than a singly infected RBC. While multiple infections of single RBCs do occur, it is unknown whether in these situation both/all parasites would produce viable merozoites. However, the possibility cannot be dismissed.

Finally, we have made several simplifying assumptions about how decreases in RBC abundance (both total and susceptible RBCs) impact on the probability of free merozoites invading a suitable RBC. The dynamics of this interaction would be best represented by considering the number of “encounters” between susceptible RBCs and free merozoites under flow conditions. Such a micro-scale model could be produced however there is currently a lack of detailed data describing specific aspects of the biological process (for example, the duration of survival of free merozoites after schizont rupture). Such data are required in order to improve the simplifying assumptions made in the current model.

In summary, we have developed a simulation model to investigate whether preferential invasion of certain host RBCs by *Plasmodium* merozoites is able to self-limit the parasite burden and protect the host from severe anaemia. It is apparent from our results that, in the absence of a specific immune response, preferential invasion can provide a mechanism by which parasite numbers can be regulated, reducing the severity of host anaemia. The model also highlights the importance of the maximum parasite replication rate, and the duration of the erythrocytic cycle, in regulating the course of an infection. Although the purpose of the parasite regulating its own growth is to maximise its chance of transmission, limiting the parasite burden of infection may be a crucial step in ensuring sufficient time is available to the host to develop an effective immune response.

## Supporting Information

Figure S1 **Under numerous scenario conditions, the total RBC population, and the parasite population reached a state of equilibrium.** This figure was generated specifying *m_max_* = 18, a 48 hour erythrocytic cycle duration, and preferential invasion of reticulocytes (RBC age class <2 days). Histograms to the right indicate changes to the RBC age class distribution through time.
(TIF)

Figure S2 **Time to equilibrium was assessed by conducting simulations for a maximum of 2000 days.** If the total RBC population, and the parasite population remained stable (to four significant figures) for 10 days, we determined that equilibrium had been reached. If equilibrium was not achieved within 2000 days, we assume the scenario has entered a cyclic state and will never reach equilibrium. In those populations where equilibrium was achieved, 2/3rds reached this state within 400 days.
(TIF)

Figure S3 **Preferential invasion of young RBCs and relative RBC density in infected hosts after 400 days.** Relative RBC density is mapped as a function of the maximum number of merozoites released per schizont, and the incrementally increasing maximum age of susceptible RBCs (simulated with an erythrocytic cycle duration of 48 hours). Darker areas indicate greater RBC loss.
(TIF)

Figure S4 **Preferential invasion of young RBCs and maximum parasitemia in infected hosts.** Maximum parasitemia is mapped as a function of the maximum number of merozoites released per schizont, and the incrementally increasing maximum age of susceptible RBCs (simulated with an erythrocytic cycle duration of 48 hours). Darker areas indicate lower peak parasitemia.
(TIF)

Figure S5 **Preferential invasion of young RBCs and time required to reach maximum parasitemia in infected hosts.** Density is mapped as a function of the maximum number of merozoites released per schizont, and the incrementally increasing maximum age of susceptible RBCs (simulated with an erythrocytic cycle duration of 48 hours). Darker areas indicate infections reach maximum parasitemia in shorter periods of time. The effects of preferential invasion are less pronounced, but more apparently as the maximum number of merozoites released per schizont increases.
(TIF)
