# Supplementary figures and images for: Preferential Invasion by Plasmodium Merozoites and the Self-Regulation of Parasite Burden
Source: PLoS One. 2013 Feb 27;8(2):e57434. doi: 10.1371/journal.pone.0057434 (PMC3584029; doi:10.1371/journal.pone.0057434)

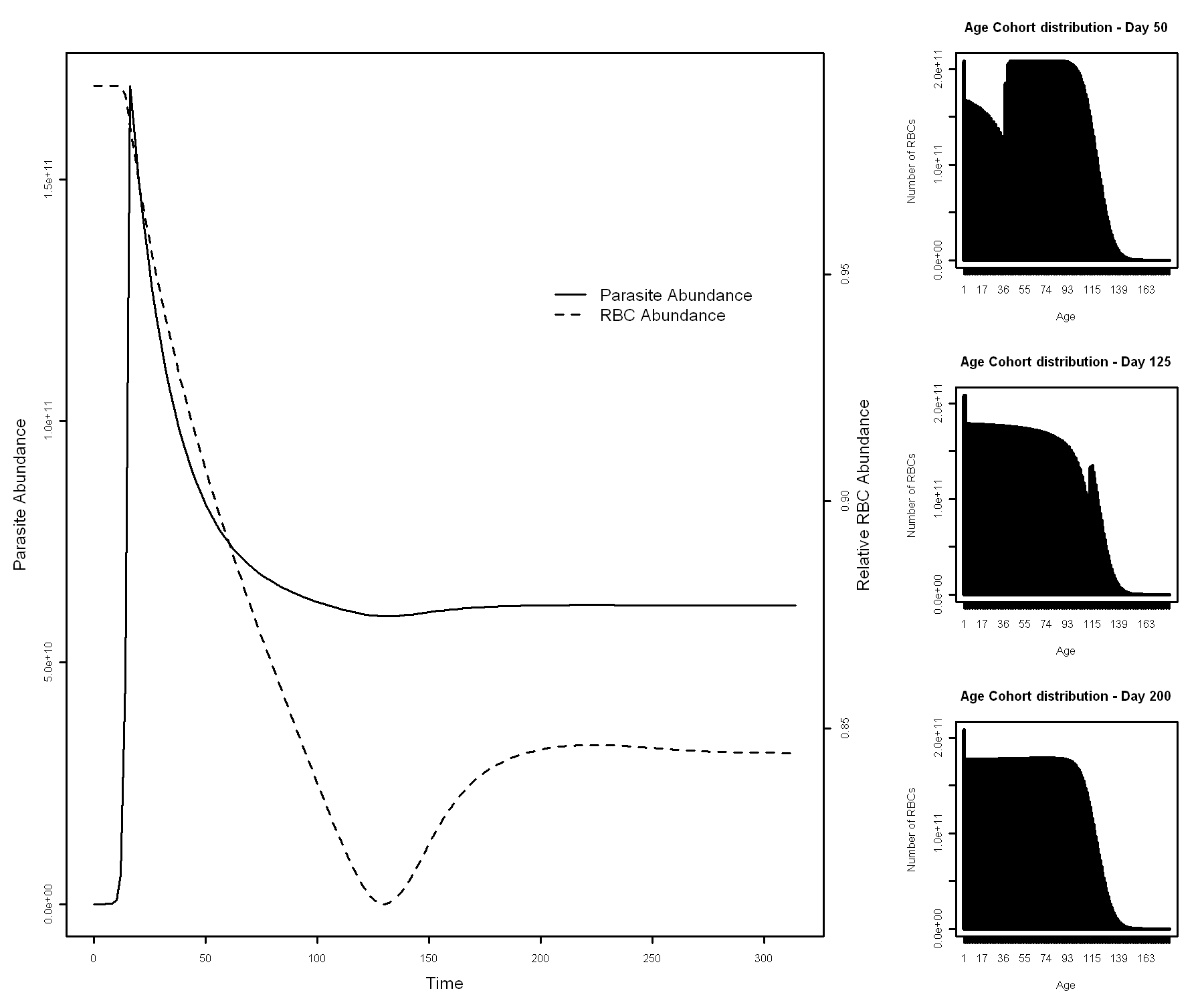

Supplement: Figure S1 — Under numerous scenario conditions, the total RBC population, and the parasite population reached a state of equilibrium. This figure was generated specifying mmax = 18, a 48 hour erythrocytic cycle duration, and preferential invasion of reticulocytes (RBC age class <2 days). Histograms to the right indicate changes to the RBC age class distribution through time. (TIF) [file pone.0057434.s001.tif]

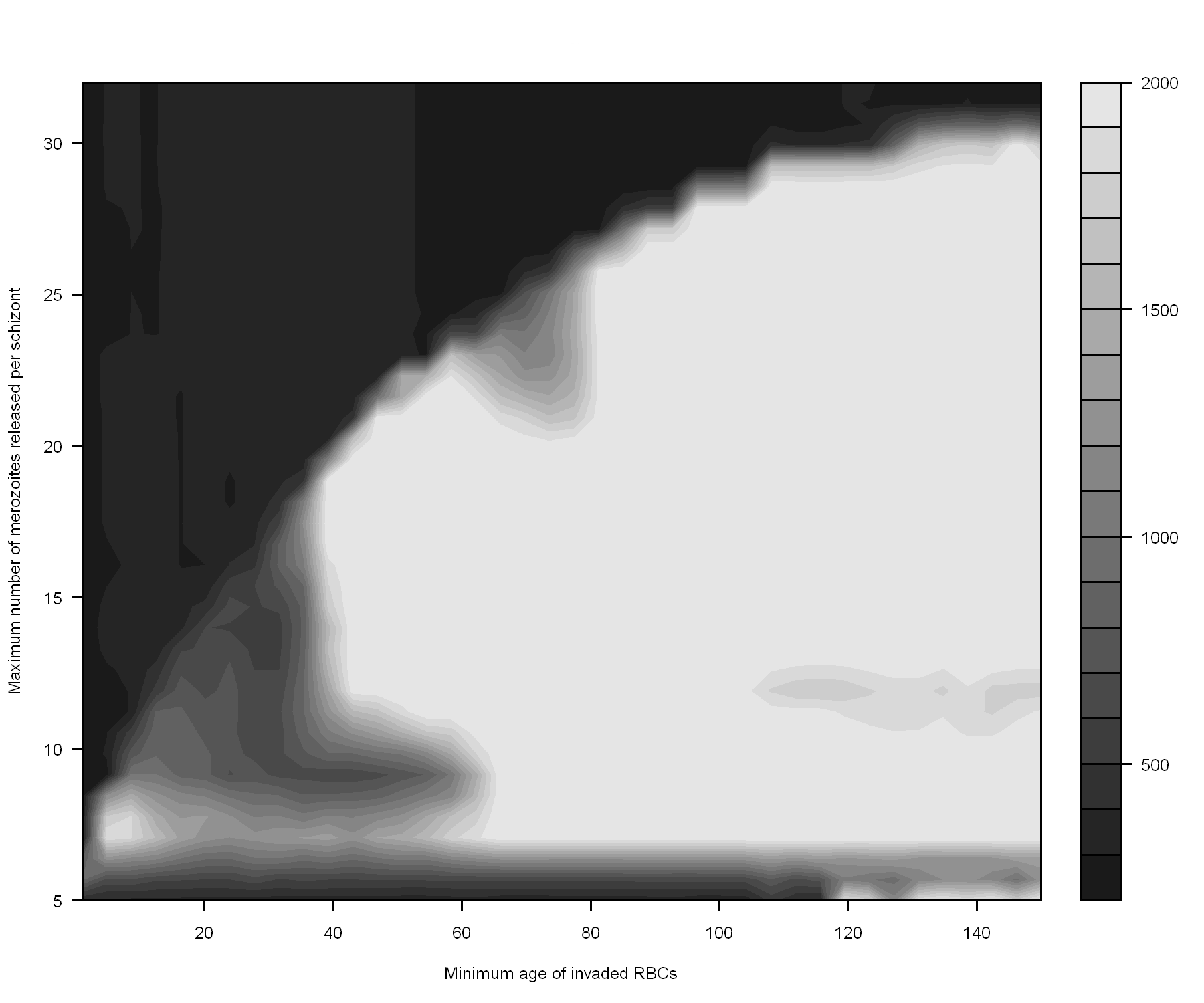

Supplement: Figure S2 — Time to equilibrium was assessed by conducting simulations for a maximum of 2000 days. If the total RBC population, and the parasite population remained stable (to four significant figures) for 10 days, we determined that equilibrium had been reached. If equilibrium was not achieved within 2000 days, we assume the scenario has entered a cyclic state and will never reach equilibrium. In those populations where equilibrium was achieved, 2/3rds reached this state within 400 days. (TIF) [file pone.0057434.s002.tif]

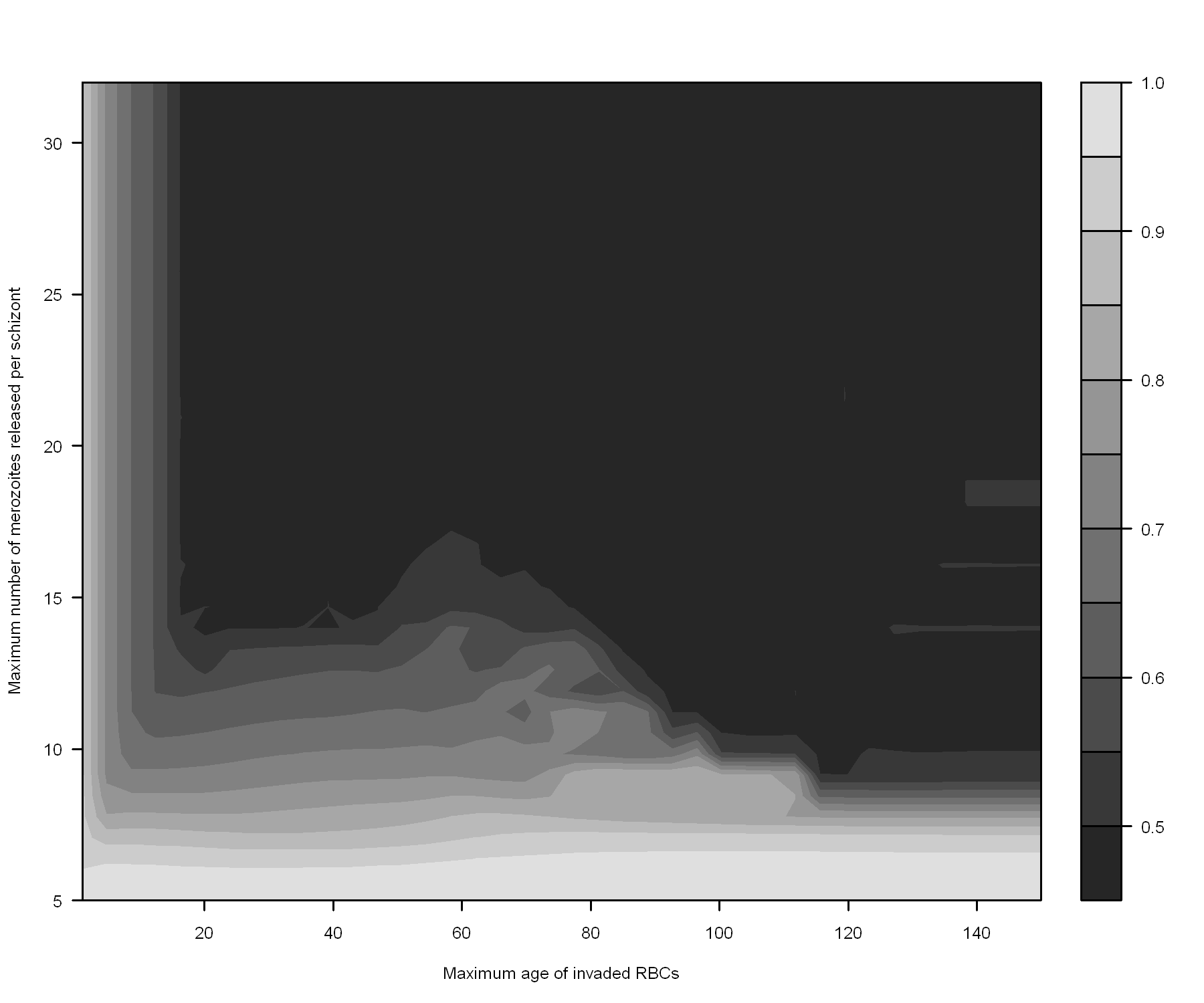

Supplement: Figure S3 — Preferential invasion of young RBCs and relative RBC density in infected hosts after 400 days. Relative RBC density is mapped as a function of the maximum number of merozoites released per schizont, and the incrementally increasing maximum age of susceptible RBCs (simulated with an erythrocytic cycle duration of 48 hours). Darker areas indicate greater RBC loss. (TIF) [file pone.0057434.s003.tif]

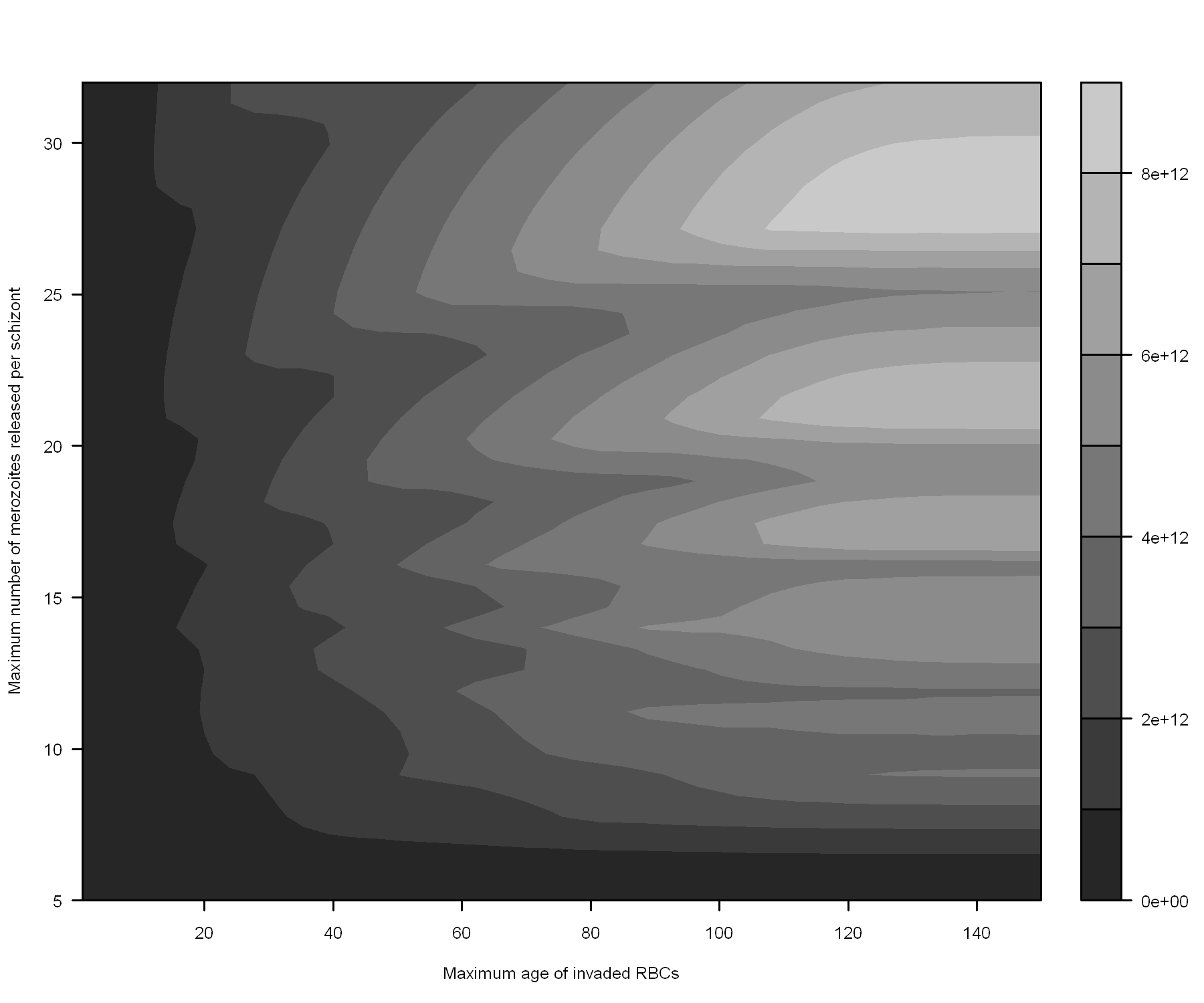

Supplement: Figure S4 — Preferential invasion of young RBCs and maximum parasitemia in infected hosts. Maximum parasitemia is mapped as a function of the maximum number of merozoites released per schizont, and the incrementally increasing maximum age of susceptible RBCs (simulated with an erythrocytic cycle duration of 48 hours). Darker areas indicate lower peak parasitemia. (TIF) [file pone.0057434.s004.tif]

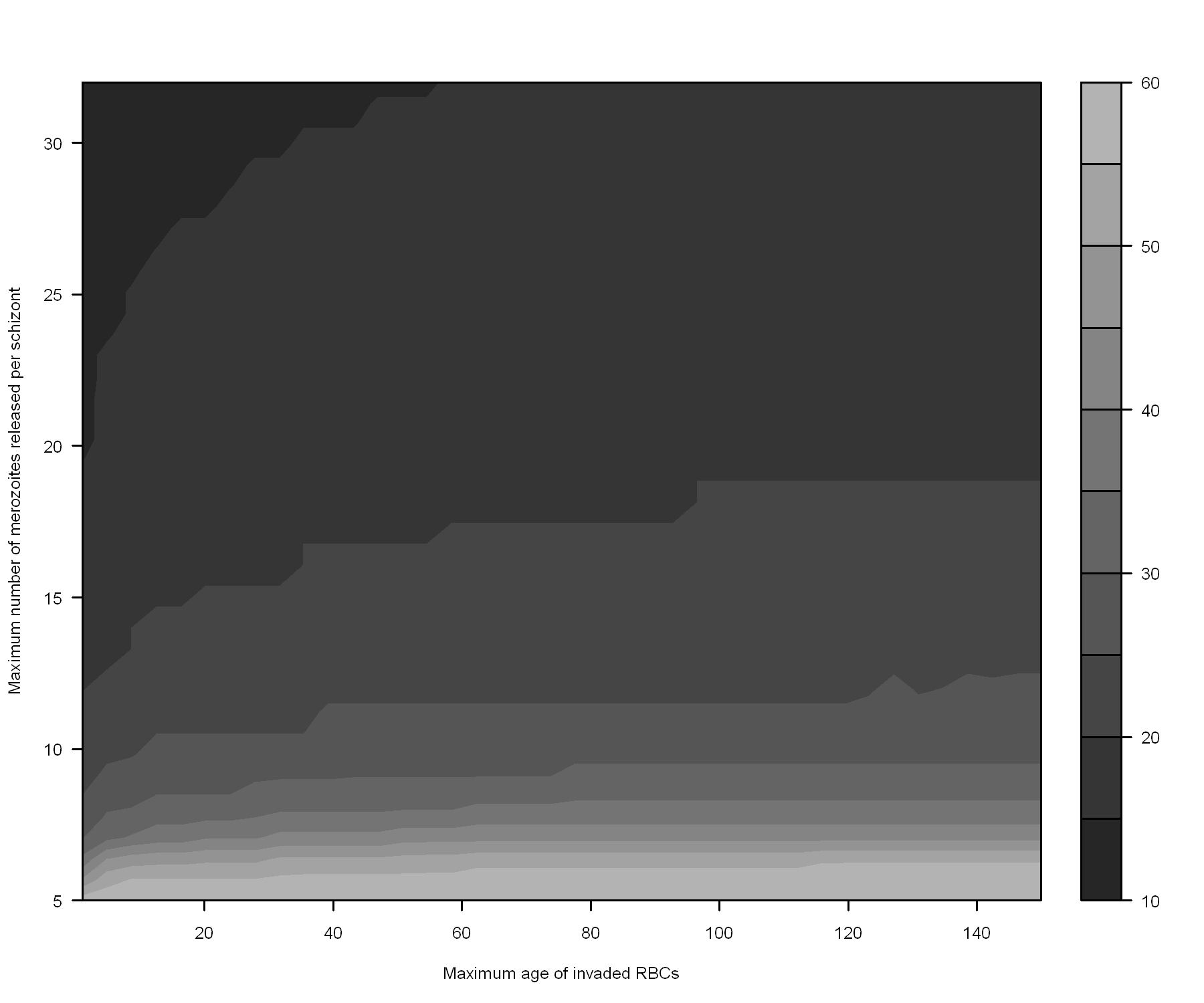

Supplement: Figure S5 — Preferential invasion of young RBCs and time required to reach maximum parasitemia in infected hosts. Density is mapped as a function of the maximum number of merozoites released per schizont, and the incrementally increasing maximum age of susceptible RBCs (simulated with an erythrocytic cycle duration of 48 hours). Darker areas indicate infections reach maximum parasitemia in shorter periods of time. The effects of preferential invasion are less pronounced, but more apparently as the maximum number of merozoites released per schizont increases. (TIF) [file pone.0057434.s005.tif]
